# Association of Serum Phytoestrogen Concentration and Dietary Habits in a Sample Set of the JACC Study

**DOI:** 10.2188/jea.15.S196

**Published:** 2005-08-18

**Authors:** Kotaro Ozasa, Masahiro Nakao, Yoshiyuki Watanabe, Kyohei Hayashi, Tsuneharu Miki, Kazuya Mikami, Mitsuru Mori, Fumio Sakauchi, Masakazu Washio, Yoshinori Ito, Koji Suzuki, Tatsuhiko Kubo, Kenji Wakai, Akiko Tamakoshi

**Affiliations:** 1 Department of Epidemiology for Community Health and Medicine, Kyoto Prefectural University of Medicine Graduate School of Medical Science.; 2 Department of Urology, Kyoto Prefectural University of Medicine Graduate School of Medical Science.; 3 Department of Public Health, Sapporo Medical University School of Medicine.; 4 Department of Public Health, Fujita Health University School of Health Sciences.; 5 Division of Epidemiology and Prevention, Aichi Cancer Center Research Institute.; 6 Department of Clinical Epidemiology, University of Occupational and Environmental Health.; 7 Department of Preventive Medicine/Biostatistics and Medical Decision Making, Nagoya University Graduate School of Medicine.; * Department of Urology, Meiji University of Oriental Medicine. (current)

**Keywords:** phytoestrogen, Isoflavones, Food Habits, validity, Cohort Studies

## Abstract

BACKGROUND: Phytoestrogens may be associated with a reduced risk of hormone dependent neoplasms such as prostate and breast cancers. We tried to determine the validity of the association between serum phytoestrogen concentrations and dietary habits obtained from a food frequency questionnaire used in the Japan Collaborative Cohort Study (JACC Study) for Evaluation of Cancer Risk sponsored by the Ministry of Education, Science, Sports and Culture of Japan (Monbusho).

METHODS: The subjects were 151 male controls who were selected for a nested case-control study for evaluating prostate cancer risk as part of the JACC Study. Dietary habits were determined using a food frequency questionnaire at baseline, and the concentrations of genistein, daidzein, and equol in frozen-stored serum samples assayed in 2002 were compared.

RESULTS: Tofu intake showed a significant association with the serum concentrations of genistein and daidzein (Spearman’s correlation coefficients (*r_s_*) =0.30 and 0.27, respectively), and miso soup showed a slight association with serum concentrations of these phytoestrogens. In contrast, serum concentrations of equol were not associated with dietary intake of tofu and miso soup. After adjustment for serum daidzein concentration, serum equol concentration was associated with the intake of foods containing fat, meat, and coffee, but not green tea.

CONCLUSIONS: Serum genistein and daidzein concentrations were significantly associated with dietary intake of tofu, and slightly with intake of miso soup. Consumption of fat, meat, and coffee may be associated with equol production by intestinal microflora in this sample set.

Phytoestrogens are isoflavonoids and lignans of plant origin with estrogen-like activities that may be associated with a reduced risk of hormone dependent neoplasms such as prostate and breast cancers.^1^^,^^2^ Consumption of large amounts of phytoestrogen-rich foods such as soybeans may be associated with the low incidence of prostate and breast cancers in Japan and other Asian countries. A nested case-control study within the Japan Collaborative Cohort Study (JACC Study) for Evaluation of Cancer Risk sponsored by the Ministry of Education, Science, Sports and Culture of Japan (Monbusho)^3^ found that a high serum concentration of phytoestrogens was associated with a reduced risk of prostate cancer.^4^

Variables evaluated at baseline of a cohort study should reflect the long-term condition of subjects. Turnover of phytoestrogens in serum is thought to be relatively rapid, with half times of about 6-8 hours.^5^ The relationship between dietary intake of phytoestrogens and their serum concentrations should therefore be investigated. In addition, equol, a strong phytoestrogen, is thought to be produced from daidzein by intestinal microflora of particular individuals,^2^ suggesting that the relationship between dietary intake of daidzein and the serum concentration of equol should therefore be examined to determine the dietary habits related to equol-producing ability.

We therefore examined the association between serum phytoestrogen concentrations and dietary habits obtained from a food frequency questionnaire used in the baseline survey of the JACC Study and tried to validate it. Additionally, some issues about equol production were discussed.

## METHODS

The subjects were 151 male controls who were selected for a nested case-control study,^4^ performed as part of the JACC Study,^3^ in which the risk of prostate cancer was related to serum phytoestrogen concentrations. The mean age of the subjects was 68.7 years old (range, 58 to 83 years).

Dietary habits of the subjects were surveyed using a self-administered food frequency questionnaire at the baseline survey of the JACC Study in 1988-90. Each subject indicated his average frequency of consumption of 32 food items; beef, pork, ham and sausage, chicken, liver, egg, milk, yogurt, cheese, butter, margarine, fried food, fried vegetables, fish (unprocessed), boiled fish paste (‘*kamaboko*’ in Japanese), dried or salted fish, green-leaf vegetables, carrots and squash, tomatoes, cabbage and lettuce, Chinese cabbage, edible wild plants (‘*sansai*’), mushroom, potatoes, seaweed, pickles, food boiled down in soy sauce, boiled beans, tofu (soybean curd), oranges, fruits other than oranges, and fruit juice. There were five frequency categories: scarcely, 1-2 times a month, 1-2 times a week, 3-4 times a week, and almost every day. Boiled rice was evaluated as the number of bowls consumed per day. Miso soup consumption was re-categorized as five levels: every other day or less, one bowl a day, two bowls a day, three bowls a day, and four or more bowls a day. Beverages such as coffee, tea, Japanese tea (including green tea, coarse tea (‘*ban-cha*’), and roasted tea (‘*hoji-cha*’)), and Chinese tea were evaluated by frequency (scarcely, 1-2 times a month, 1-2 times a week, 3-4 times a week, and almost every day), with the number of cups a day recorded for those consuming these beverages almost every day.

Each subject donated blood samples at health-screening checks near the baseline survey.^3^ Serum was obtained and stored at -80°C until analyzed. Individual written or oral consent, or consent from community representatives, was obtained, or poster notification/opting-out system was applied.^3^ The Ethical Boards of Nagoya University School of Medicine and the Kyoto Prefectural University of Medicine approved this study.

All serum samples were assayed at a single laboratory (SRL, Hachioji, Japan) in 2002, with the staff blinded to case-control status. Serum levels of daidzein, genistein, and equol were measured as described.^6^ Briefly, each sample was hydrolyzed and incubated overnight at 37°C with *β*-glucuronidase/sulfatase (Nippon Biotest Laboratories Inc., Kokubunji, Japan) in acetate buffer (pH 4.5). The dimethyl ether extract of the sample was dried under nitrogen flow and redissolved in a 2:1:3 mixture of methanol, acetonitrile and water. Each sample was centrifuge-filtered using Ultrafree-MC, 0.22 *μ*m pore size (Millipore, Bedford, MA), and the concentrations of daidzein, genistein, and equol were measured using an LC/MS/MS system (LC: HP1100 Series, Agilent Technologies, Palo Alto, CA, MS/MS: Quattro-Ultima, Micromass Ltd, Manchester, UK). The ionizing method was electrospray using negative ions, and multiple reaction monitoring was used for mass analysis. Reference genistein, daidzein, and equol were made by Extrasynthese S.A. (Genay Cedex, France). For quality control, we determined the variation of measurements for two samples. We found that the coefficients of variation (CV) were 6.5% and 7.5% for genistein, 6.9% and 8.2% for daidzein, and 8.2% and 9.1% for equol. Equol-producers were defined as subjects whose serum equol was detected (>6.9 nM) in this study.

Geometric means of serum phytoestrogen concentrations were compared with levels of food intake because concentrations of the former were log-normally distributed. Pearson’s correlation coefficient (*r*) between serum phytoestrogen concentrations was calculated for log-transformed values of moles per liter.

The association between serum phytoestrogen concentrations and food intake was examined using Spearman’s correlation coefficients (*r_s_*) and regression coefficients (*b*), which were calculated using integral numbers for food intake levels and log-transformed data. We also attempted to estimate serum phytoestrogen concentration by a multivariate regression model.

Geometric means of daidzein concentration between equol-producers and nonequol-producers were compared using Student’s t-test, and their relationship to the intake of various foods was compared by Wilcoxon’s rank test. Food intake related to production of equol was examined using Spearman’s partial correlation coefficients after adjustment for serum daidzein concentrations, with serum equol concentration set at zero for nonequol-producers. We assumed that p values less than 0.05 as statistically significant, and also mentioned tendencies with p values less than 0.1 for discussion.

## RESULTS

**Figure 1.**
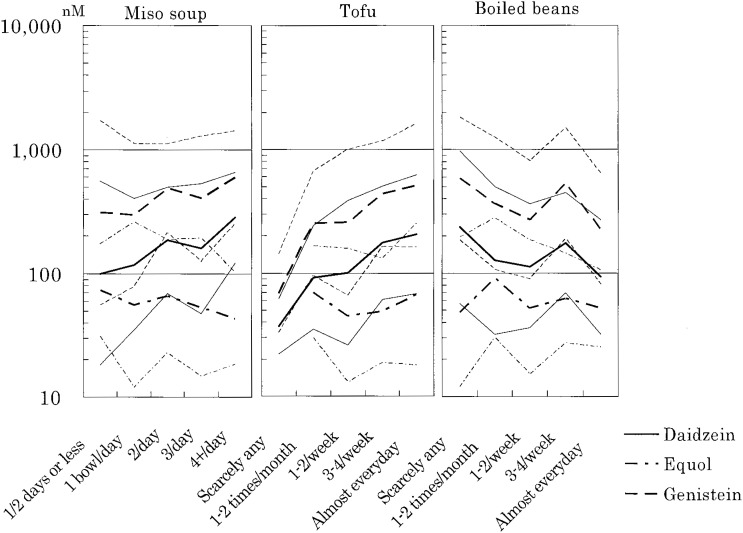
Association of food intake and serum phytoestrogen concentration. Bold lines show the geometric means of each category of food intake. Thin lines show the mean ± SD of log-transformed data.

The geometric mean concentrations for all subjects were 368 nM for genistein (mean ± standard deviation of log-transformed data; 111, 1222 nM), 139 mM for daidzein (40, 477 nM), and 55 nM for equol for equol producers (16, 187 nM). Figure 1 shows the relationship between serum concentrations of these phytoestrogens and food intake, and Table 1 shows the parameters of association (*r_s_*, *b*). Tofu intake was significantly associated with serum concentrations of genistein and daidzein (coefficients of determination (R^2^) = 0.081 and 0.082, respectively). Intake of miso soup was almost significantly associated with daidzein concentration. Multivariate regression modeling with tofu and miso soup intake slightly reduced the R^2^ for genistein (0.065) and daidzein (0.074). Intake of boiled beans was not associated with serum concentrations of either. In determining the relationship between dietary intake and equol concentration, we calculated *r_s_* for all subjects and limited *b* to equol-producers. We found that serum concentration of equol was associated with intake of tofu among all subjects.

**Table 1.** Spearman’s correlation coefficients and regression coefficients of food intake for phytoestrogens.

Food item	Genistein		Daidzein		Equol^†^	
		
	Spearman’s correlated coefficient	Regression coefficient	Spearman’s correlated coefficient	Regression coefficient	Spearman’s correlated coefficient	Regression coefficient
Miso-soup	0.09	0.14	0.16^+^	0.19*	0.03	-0.07
Tofu	0.30**	0.34**	0.27**	0.35**	0.21*	0.12
Boiled beans	-0.11	-0.11	-0.12	-0.11	-0.04	-0.07

+ : p<0.1, *: p<0.05, **: p<0.01.

† : Spearman’s correlated coefficient was calculated for all subjects, and regression coefficient was limited to equol-products.

**Figure 2.**
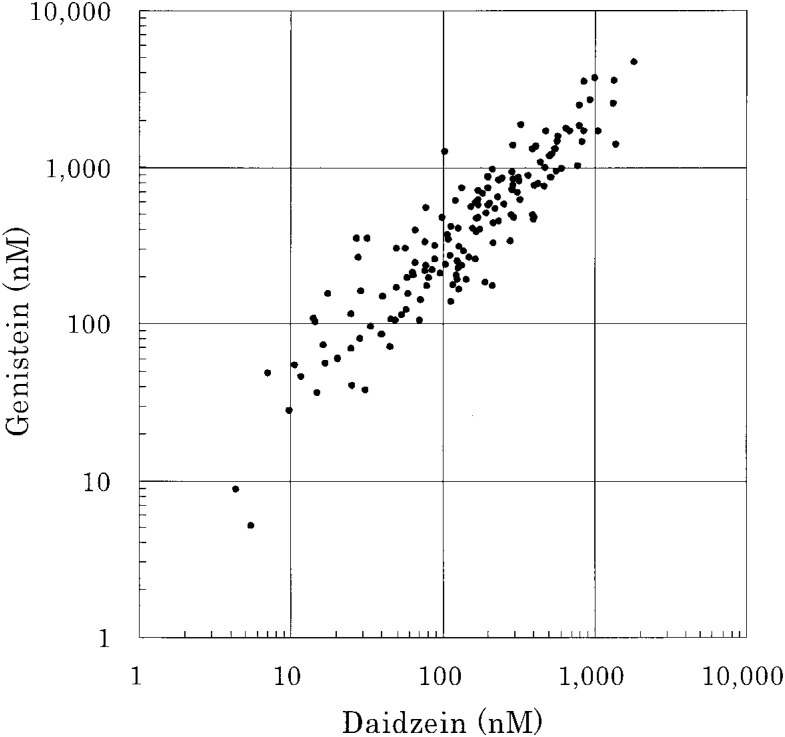
Correlation between serum daidzein and genistein concentrations.

**Figure 3.**
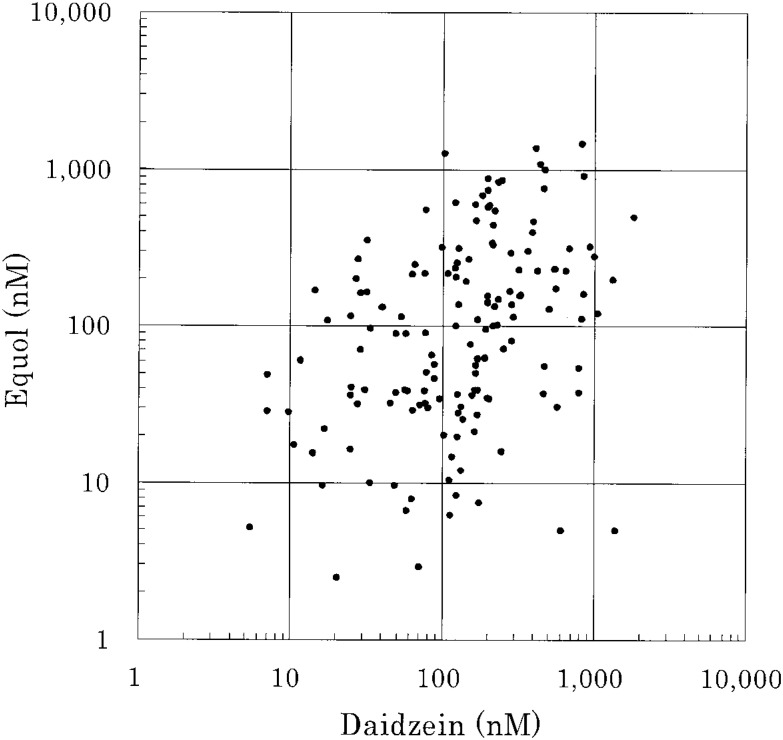
Correlation between serum daidzein and equol concentrations among equol-producers.

Serum genistein and daidzein concentrations were strongly correlated (*r* = 0.91, p = 0.0001, Figure 2). Serum daidzein and equol concentrations among equol producers were moderately correlated (*r* = 0.40, p = 0.0001, Figure 3). Among the 113 equol producers, the geometric mean concentrations of daidzein were 142 nM for equol-producers (n = 113) and 137 nM for nonequol-producers (n = 38), and they did not differ (p = 0.90). Foods consumed significantly by equol-producers than by nonequol-producers included pork (p = 0.0014 by Wilcoxon’s test), margarine (p = 0.019), Chinese cabbage (p = 0.034), and tofu (p = 0.037), and nearly significant for milk (p = 0.051), *Kamaboko* (p = 0.099), and seaweed (p = 0.056).

Foods showing a significant association with serum equol concentration after adjustment for serum daidzein concentration included pork, butter, margarine, cheese, coffee, ham, and seaweeds, and nearly significant for tofu, potatoes, and milk. Those Spearman’s partial correlation coefficients are listed in Table 2.

**Table 2.** Spearman’s partial correlation coefficients between serum equol concentration and frequency of food intake adjusted for serum daidzein concentration.

Food	Spearman’s partial correlation coefficients
Pork	0.34 **
Butter	0.31 **
Margarine	0.29 **
Cheese	0.29 **
Coffee	0.19 *
Ham	0.18 *
Seaweeds	0.17 *
Tofu	0.17 +
Potatoes	0.15 +
Milk	0.14 +

+: p<0.1, *: p<0.05, **: p<0.01.

Food list is arranged in order of p value.

## DISCUSSION

This study was primarily designed to validate the food frequency questionnaire and serum concentrations of phytoestrogens in the nested case-control study as a part of the JACC Study.^4^ Controls of the nested case-control study were thought to represent the general population of the JACC study, and adequate to the sample set of this study. However, additional discussion such as about equol producing may be limited; e.g., most subjects of the JACC Study lived in rural areas, where dietary habits may be different from those in urban areas; subjects who donated blood samples were around 30% of whole respondents to the baseline questionnaire survey, therefore, they may be more health-conscious.

Quantitative stability of phytoestrogens during long-term storage at -80°C is not established, so we assumed it from the viewpoint of molecular structure of isoflavones. Ranges of those serum levels in this study were comparable to those found in previous studies in Japan.^5^^,^^7^^,^^9^

We have shown here that serum concentrations of genistein and daidzein were each associated with dietary tofu intake, and slightly associated with miso soup intake. Genistein and daidzein are contained mainly in soybeans, the main ingredients of tofu and miso soup. Dietary habits of tofu and miso soup intake can be clearly evaluated in the general population, such as in the male subjects of this study, thus showing clear associations. The dietary category of boiled beans includes beans other than soybeans, and it may be difficult for study subjects to evaluate their intake of this food. We were therefore unable to determine an association between dietary intake of boiled beans and serum concentrations of phytoestrogens. Natto (fermented soybeans), one of the main foods containing soybeans in Japan, was not listed in the baseline survey questionnaire.

It is meaningful that an association was shown between longterm intake of soybean products and the concentrations of genistein and daidzein in a one-point serum sample despite the short half-lives of these phytoestrogens (6-8 hrs).^5^ Frequent intake of phytoestrogen-rich foods may be required to maintain their blood concentration. An unusually large intake of these foods prior to taking a blood sample may result in unusually high blood concentrations of phytoestrogens, whereas abstention from these foods for long periods of time may result in unusually low blood concentrations of phytoestrogens. Our results show that these events were not frequent enough to diminish the association between dietary intake and phytoestrogen concentration, suggesting that dietary intake of these foods is stable. The absence of natto from the list of foods, however, may explain, at least in part, the low validity of association observed in this study.

Validity studies of serum phytoestrogen concentrations with respect to food intake have been scarce in Japan. One study reported that Spearman’s correlation coefficients of serum daidzein concentration with dietary intake of natto, miso, and tofu ranged from 0.19 to 0.23, whereas the correlation coefficient of serum daidzein concentration with daidzein intake estimated from dietary records was 0.37 and with daidzein intake estimated by a food frequency questionnaire was 0.26.^7^ Our results were quite consistent with these earlier findings.

Equol is produced by intestinal microflora and absorbed.^2^ We observed no differences in serum daidzein concentrations between equol-producers and nonequol-producers, suggesting that the ability to produce equol did not depend on daidzein intake. Among equol-producers, we did observe a slight dependence of serum equol concentration on serum daidzein concentration. Equol excretion has been reported to correlate positively with the intake of total fat and meat, and the fat-fiber ratio,^2^^,^^8^ suggesting that consumption of more fat and meat creates a colonic environment capable of sustaining equol-producing microflora. It is also possible, however, that equol is contained in the meat of animals consuming soy-, alfalfa-, or clover-supplemented feed.^2^ Our results indicate that consumption of fat and meat is associated with serum equol concentrations. Among Japanese people, who have a lower fat and meat intake than Western people, the effect of fat and meat on serum equol concentration may be clearer.

Coffee consumption was also correlated with serum equol concentration, although intake of other beverages, including various teas, did not show this association. The correlation between coffee consumption and serum equol concentration was still observed after adjustment for intake of pork, cheese, and butter (*r_s_* = 0.21, p = 0.078). Residual confounding should be considered. That is, coffee consumption may be associated with a Western diet, which involves the consumption of more fat and meat than a Japanese diet. Another Japanese study showed that green tea consumption was higher among equol-producers than nonequol-producers, but the two groups showed no differences in coffee and tea consumption.^9^ Our study, however, did not show this relationship (*r_s_* = -0.11, p = 0.22 for Japanese tea).

When we examined the difference in food intake between equol-producers and nonequol-producers, we found that equol producers consumed significantly higher quantities of some foods, including pork, margarine, Chinese cabbage, and tofu. This was similar to the results of correlation analysis. We regard the latter as more valid because it took serum equol concentration into account when considering serum daidzein concentration.

In conclusion, we have shown here that serum genistein and daidzein concentrations were significantly associated with dietary intake of tofu, and slightly with intake of miso soup. Therefore, food frequency questionnaire for soy bean products and serum phytoestrogen concentrations seemed to be valid in the JACC Study. Consumption of fat, meat, and coffee may be associated with equol production by intestinal microflora in the sample set of this study.

## MEMBER LIST OF THE JACC STUDY GROUP

The present investigators involved, with the co-authorship of this paper, in the JACC Study and their affiliations are as follows: Dr. Akiko Tamakoshi (present chairman of the study group), Nagoya University Graduate School of Medicine; Dr. Mitsuru Mori, Sapporo Medical University School of Medicine; Dr. Yutaka Motohashi, Akita University School of Medicine; Dr. Ichiro Tsuji, Tohoku University Graduate School of Medicine; Dr. Yosikazu Nakamura, Jichi Medical School; Dr. Hiroyasu Iso, Institute of Community Medicine, University of Tsukuba; Dr. Haruo Mikami, Chiba Cancer Center; Dr. Yutaka Inaba, Juntendo University School of Medicine; Dr. Yoshiharu Hoshiyama, University of Human Arts and Sciences; Dr. Hiroshi Suzuki, Niigata University School of Medicine; Dr. Hiroyuki Shimizu, Gifu University School of Medicine; Dr. Hideaki Toyoshima, Nagoya University Graduate School of Medicine; Dr. Kenji Wakai, Aichi Cancer Center Research Institute; Dr. Shinkan Tokudome, Nagoya City University Graduate School of Medical Sciences; Dr. Yoshinori Ito, Fujita Health University School of Health Sciences; Dr. Shuji Hashimoto, Fujita Health University School of Medicine; Dr. Shogo Kikuchi, Aichi Medical University School of Medicine; Dr. Akio Koizumi, Graduate School of Medicine and Faculty of Medicine, Kyoto University; Dr. Takashi Kawamura, Kyoto University Center for Student Health; Dr. Yoshiyuki Watanabe, Kyoto Prefectural University of Medicine Graduate School of Medical Science; Dr. Tsuneharu Miki, Graduate School of Medical Science, Kyoto Prefectural University of Medicine; Dr. Chigusa Date, Faculty of Human Environmental Sciences, Mukogawa Women’s University ; Dr. Kiyomi Sakata, Wakayama Medical University; Dr. Takayuki Nose, Tottori University Faculty of Medicine; Dr. Norihiko Hayakawa, Research Institute for Radiation Biology and Medicine, Hiroshima University; Dr. Takesumi Yoshimura, Fukuoka Institute of Health and Environmental Sciences; Dr. Akira Shibata, Kurume University School of Medicine; Dr. Naoyuki Okamoto, Kanagawa Cancer Center; Dr. Hideo Shio, Moriyama Municipal Hospital; Dr. Yoshiyuki Ohno, Asahi Rosai Hospital; Dr. Tomoyuki Kitagawa, Cancer Institute of the Japanese Foundation for Cancer Research; Dr. Toshio Kuroki, Gifu University; and Dr. Kazuo Tajima, Aichi Cancer Center Research Institute.
